# Translational control in plant antiviral immunity

**DOI:** 10.1590/1678-4685-GMB-2016-0092

**Published:** 2017-02-13

**Authors:** João Paulo B. Machado, Iara P. Calil, Anésia A. Santos, Elizabeth P.B. Fontes

**Affiliations:** 1 Department of Biochemistry and Molecular Biology, BIOAGRO, National Institute of Science and Technology in Plant-Pest Interactions, Universidade Federal de Viçosa, 36571.000, Viçosa, MG, Brazil.; 2 Department of General Biology, Universidade Federal de Viçosa, 36571.000, Viçosa, MG, Brazil

**Keywords:** Translation suppression, recessive resistance genes, Argonaute, NSP-Interacting Kinase, NIK

## Abstract

Due to the limited coding capacity of viral genomes, plant viruses depend extensively on the host cell machinery to support the viral life cycle and, thereby, interact with a large number of host proteins during infection. Within this context, as plant viruses do not harbor translation-required components, they have developed several strategies to subvert the host protein synthesis machinery to produce rapidly and efficiently the viral proteins. As a countermeasure against infection, plants have evolved defense mechanisms that impair viral infections. Among them, the host-mediated translational suppression has been characterized as an efficient mean to restrict infection. To specifically suppress translation of viral mRNAs, plants can deploy susceptible recessive resistance genes, which encode translation initiation factors from the eIF4E and eIF4G family and are required for viral mRNA translation and multiplication. Additionally, recent evidence has demonstrated that, alternatively to the cleavage of viral RNA targets, host cells can suppress viral protein translation to silence viral RNA. Finally, a novel strategy of plant antiviral defense based on suppression of host global translation, which is mediated by the transmembrane immune receptor NIK1 (nuclear shuttle protein (NSP)-Interacting Kinase1), is discussed in this review.

## Introduction

Due to their sessile nature, plants are constantly exposed to extreme adverse conditions that affect negatively their growth and development, thereby resulting in considerable yield losses worldwide. Among the biotic factors, virus infections are one of the most prevalent agricultural constraints as they often suppress the plant defenses and severely limit productivity of relevant crops, representing a serious threat to global food security. As obligatory intracellular parasites, plant viruses depend on the host cell machinery to replicate their genome, express their genes and invade their hosts. Thus, in order to establish a productive infection, compatible interactions between viral and host factors must occur, from the expression and replication of the viral genome until the cell-to-cell movement and long distance translocation through the vascular system of viral particles. In addition to providing basic compatibility, interactions between viral and host proteins are necessary to modulate the viral infection, preventing or neutralizing the plant defense mechanisms.

Plants deploy several strategies to defend themselves against viral infections; the best characterized are the expression of resistance genes and RNA silencing (Nicaise, 2014). Naturally occurring resistance genes, which show dominant or recessive inheritance, bestow an efficient barrier to viral infection (Robaglia and Caranta, 2006). Most of the dominant resistance genes (R genes) identified in plant–virus interactions belong to the nucleotide binding site leucine-rich repeat (NBS-LRR) class, which specifically recognize viral avirulence (avr) gene products (De Ronde *et al.*, 2014; Galvez *et al.*, 2014; Nicaise, 2014). Frequently, the R protein activation elicits a hypersensitive response (HR), which is often associated with programmed cell death of infected and adjacent cells, confining the pathogen within the local site of infection (Gururani *et al.*, 2012; Galvez *et al.*, 2014). The downstream events of R protein activation might be also associated with other signals like influx of Ca^2+^ ions from the extracellular space and/or anion flux, MAPK-mediated signaling, production of reactive oxygen species (ROS), salicylic acid (SA) accumulation, extensive transcriptional reprogramming and activation of defense responses (Gururani *et al.*, 2012; Nicaise, 2014). Additionally to dominant R gene–related resistance responses, recessive resistance has been commonly reported in viral systems as another evolving strategy to impair virus infection (Kang *et al.*, 2005b). Instead of triggering typical defense responses, like the hypersensitive response, most recessive mutations lead to a non-permissive environment due to the lack of appropriate host factors, which are required for the viruses to complete their biological cycle (Ritzenthaler, 2005; Galvez *et al.*, 2014). In contrast to dominant resistance, recessive resistance seems to be more durable because the viruses can only overcome the host resistance response by adapting themselves to the missing factors (Ritzenthaler, 2005; Truniger and Aranda, 2009). Because viruses do not encode translational functions and depend exclusively on the host cell machinery to synthesize the viral proteins, it is not surprising that a large number of recessive resistance genes have been mapped to mutations in translation initiation factors (eIFs) belonging to the eIF4E and eIF4G family or their isoforms eIF(iso)4E and eIF(iso)4G (Truniger and Aranda, 2009; Wang and Krishnaswamy, 2012; Julio *et al.*, 2015). In general, these mutations prevent the interactions between host factors and viral RNAs and/or proteins, which otherwise would recruit the host apparatus of translation for the synthesis of viral proteins. The partial functional redundancy of isoforms from the eIF4E and eIF4G families allows loss-of-function mutations of one isoform to provide virus resistance without compromising the general growth performance of the plant.

RNA silencing also represents a well-documented antiviral mechanism in plants (Ding, 2010; Pumplin and Voinnet, 2013; Csorba *et al.*, 2015; Ghoshal and Sanfaçon, 2015). Viral RNAs can be addressed for degradation via the endonucleolytic activity of argonaute (AGO), the catalytic component of the RNA-induced silencing complex (RISC; Mallory and Vaucheret, 2010). In addition to the endonucleolytic cleavage, recent evidence has demonstrated that the mechanism of antiviral RNA silencing also operates by suppressing viral mRNA translation (Ghoshal and Sanfaçon, 2015). As a virulence strategy, plant viruses have evolved mechanisms to prevent RNA silencing-mediated defense, predominantly by synthesizing silencing suppressors (Bologna and Voinnet, 2014; Carbonell and Carrington, 2015).

In addition to translational defense mechanisms based on recessive resistance and RNA silencing, a novel strategy of translational suppression in plant defense against DNA viruses (begomoviruses) has recently emerged as a new paradigm of antiviral defenses in plants. In this case, the activation of the transmembrane immune receptor NIK1 [nuclear shuttle protein (NSP)-interacting kinase 1] promotes the down-regulation of translational machinery-associated genes, culminating in the inhibition of viral and host mRNAs translation, which causes an increase in tolerance to begomoviruses (Zorzatto *et al.*, 2015). These defense strategies against viruses strengthen the argument that the inhibition of translation of viral proteins or their capacity to interact with translational factors offer promising alternatives to control viruses in plants.

## Recessive resistance genes in translational control

Plants respond to pathogens through an elaborate network of genetic interactions and the outcome of these interactions can result in disease or resistance. Among the plant resistance genes, the recessive ones play relevant roles in plant defense against viruses and comprise about one-half of known antiviral resistance genes (Sanfaçon, 2015). Recessive resistance is frequently associated with the lack of host factors necessary for the completion of the virus biological cycle (Galvez *et al.*, 2014). In order to achieve a successful infection, the viruses not only need unrestricted access to the host translation machinery to synthesize their proteins, but they also need to suppress host innate defenses, which may act to impair the protein production capacity of the infected cells (Walsh and Mohr, 2011). The majority of recessive genes involved in plant–virus interactions encode eukaryotic translation initiation factors (eIFs) of the 4E or 4G family, mainly eIF4E, eIF4G and their isoforms (Kang *et al.*, 2005b; Truniger and Aranda, 2009; Wang and Krishnaswamy, 2012). The involvement of eIF4E and eIF4G was firstly reported in potyvirus infection and subsequently expanded to include other plant virus families, such as bymoviruses, cucumoviruses, ipomoviruses, sobemoviruses, carmoviruses, and waikiviruses, suggesting that they contribute to a broad mechanism of plant susceptibility to viruses (Nicaise, 2014). In eukaryotes, mRNA translation is predominantly cap-dependent and involves the assembly of an mRNA-protein complex by different eIFs (Aitken and Lorsch, 2012; Hinnebusch, 2014). eIF4E is a cap-binding protein involved in the initiation of translation, being part of the protein complex known as eIF4F, which also contains eIF4G and the DEAD-box RNA helicase eIF4A. The eIF4F complex, comprising eIF4G, eIF4E and eF4A, binds poly(A)-binding protein (PABP) and eIF3 (Jackson *et al.*, 2010; Sanfaçon, 2015). In contrast to other eukaryotes, plants possess a second form of eIF4F, named eIF(iso)4F, which includes eIF(iso)4E and eIF(iso)4G (Patrick and Browning, 2012; Sanfaçon, 2015). eIF(iso)4F has complementary activities with eIF4F, but their respective components are differentially expressed, suggesting that they may also display distinct functions (Wang, 2015). Several resistance genes encoding a mutated form of eIF4E or eIF(iso)4E proteins have been shown to mediate resistance against viral infection in a range of plant/virus interactions. These include sbm-1 against *Pea seed-borne mosaic viru*s (PsbMV) and cyv2 against *Clover yellow vein virus* (ClYVV) in pea; mo1(1), mo1(2) in lettuce against *Lettuce mosaic virus* (LMV); pvr1, pvr2 and pvr6 in pepper against *Tobacco etch virus* (TEV), *Potato virus Y* (PVY), *Pepper veinal mottle virus* (PVMV); rym4 and rym5 in barley against *Barley yellow mosaic virus* (BaYMV); nsv in melon against *Melon necrotic spot virus* (MNSV); pot-1 in tomato against PVY and TEV; lsp1 in Arabidopsis against *Turnip mosaic virus* (TuMV) and TEV (Lellis *et al.*, 2002; Ruffel *et al.*, 2002, 2005, 2006; Nicaise *et al.*, 2003; Gao *et al.*, 2004a,b; Kang *et al.*, 2005a; Stein *et al.*, 2005; Albar *et al.*, 2006; Nieto *et al.*, 2006; Andrade *et al.*, 2009). Additionally, genes encoding a mutated form of eIF4G or its defective isoforms, such as rymv1 and tsv1, are responsible for resistance to *Rice yellow mottle virus* (RYMV) and *Rice tungro spherical virus* (RTSV) in rice (Albar *et al.*, 2006; Lee *et al.*, 2010). The cum1 and cum2 mutations, coding for translation initiation factors 4E and 4G, respectively, inhibit *Cucumber mosaic virus* (CMV) multiplication and *Turnip crinkle virus* (TCV) in Arabidopsis (Yoshii *et al.*, 2004).

**Figure 1:**
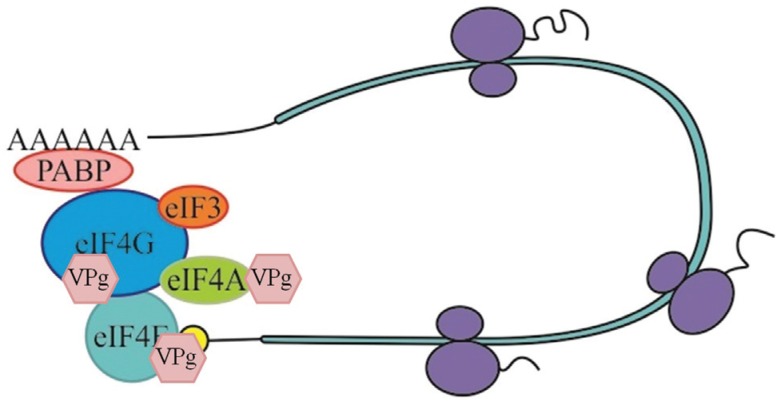
Host translation factors targeted by genome-linked viral protein (VPg) in canonical eukaryotic translation. VPg recruits the translation initiation complex for viral genome translation through its specific interaction with eIF4E/eIF(iso)4E. VPg also binds to eIF4G/eIF(iso)4G, which may act enhancing VPg-eIF4E/eIF(iso)4E interactions. Potyvirus VPg protein interacts with DEAD-box RNA helicase-like proteins closely related to *A. thaliana* eIF4A genes. RNA helicase, as part of the eIF4F translation complex, might be also involved in viral genome replication.

An important step on the elucidation of the molecular nature of recessive resistance was the identification of VPg (genome-linked viral protein) from several potyviruses as an interacting partner of the translation initiation factor 4E (eIF4E) or its isoform eIF(iso)4E in yeast two-hybrid and *in vitro* binding assays (Figure 1) (Wittmann *et al.*, 1997; Léonard *et al.*, 2000; Schaad *et al.*, 2000; Lellis *et al.*, 2002; Robaglia and Caranta, 2006). Mutations in VPg, which disrupt VPg-eIF(iso)4E interaction, impair viral infection *in planta* (Léonard *et al.*, 2000). The VPg protein may act mimicking the 5’-cap structure of messenger RNAs and recruiting the translation complex for viral genome translation through its specific interaction with eIF4E/eIF(iso)4E (Michon *et al.*, 2006; Wang, 2015). Thus, VPg protein facilitates viral RNA translation by competing with the eIF4E/eIF(iso)4E cap binding activity and enhancing the affinity of eIF4E/eIF(iso)4E for viral RNAs *in vitro.* (Plante *et al.*, 2004; Khan *et al.*, 2008). The host factor eIF4G/eIF(iso)4G is also important for a potyvirus infection as it enhances VPg-eIF4E/eIF(iso)4E interactions (Nicaise *et al.*, 2007). The VPg protein of *Rice yellow mottle virus* (RYMV) binds directly to eIF(iso)4G, rather than to eIF4E isoforms (Hebrard *et al.*, 2010). These observations suggest that VPg recruits the whole eIF4F complex, possibly for the translation of viral RNAs (Sanfaçon, 2015).

Although the VPg-eIF4E/iso4E complex may support viral RNA translation in some potyvirus-host interactions, it probably contributes to other steps of the infection. Accordingly, *Tobacco etch virus* (TEV) has been demonstrated to depend on eIF(iso)4E for its systemic spread in Arabidopsis, suggesting a role of eIF(iso)4E in viral movement (Contreras-Paredes *et al.*, 2013). This interpretation was consistent with the observation that inactivation and overexpression of the *eIF(iso)4E* gene did not affect global cellular or viral translation and, in the null mutant, viral replication was still observed. These results indicate that, for the TEV-Arabidopsis system, eIF(iso)4E is not required for viral translation and replication in the viral infective cycle, but is required for viral movement, as TEV systemic spread was completely abolished in the *eif(iso)4f* knockout lines.

Other components of the host translation machinery, such as eEF1A and PABP, are found in the virus translation/replication complex (Beauchemin and Laliberte, 2007; Thivierge *et al.*, 2008; Wang, 2015). eEF1A, eEF1B and an eIF3 subunit are required for TMV infection (Osman and Buck, 1997; Yamaji *et al.*, 2006; Hwang *et al.*, 2013). The RNA of *Turnip yellow mosaic virus* (TYMV) possesses a tRNA-like structure at 3’ UTR instead of a poly(A) tail, which works as a translation enhancer of the viral RNA. This region interacts with the host factor eEF1A and seems to regulate viral replication (Matsuda and Dreher, 2004; Sanfaçon, 2015). The ribosomal protein P0 has been correlated with viral RNA translation in *Potato virus A* (PVA) infection (Hafren *et al.*, 2013). Huang *et al.* (2010) have reported the identification of two VPg-interacting plant DEAD-box-containing RNA helicase-like proteins, AtRH8 from Arabidopsis and PpDDXL from peach (Figure 1). These proteins share sequence homology with eIF4A, a component of the eIF4F multiprotein complex. AtRH8 is not required for plant growth and development, but is necessary for viral infection. Arabidopsis *atrh8* mutant plants were resistant to both plant potyviruses *Plum pox virus* (PPV) and TuMV.

Additionally to their roles in the host translation of mRNAs, translation factors play other biological functions that might be exploited by viruses. eIF4E has been shown to accumulate in nuclear bodies, where it is involved in the export of a subset of mRNAs containing a structure known as a 4E-sensitivity element (Goodfellow and Roberts, 2008, Truniger and Aranda, 2009). In this context, it would be possible that VPg acts in the nucleus suppressing eIF4E-mediated mRNA exportation to the cytoplasm (Wang and Krishnaswamy, 2012). This hypothesis is supported by the observation that VPg inhibits the translation of the capped mRNAs (Khan *et al.*, 2008; Eskelin *et al.*, 2011). eIF4E–VPg complex may be also involved in the suppression of RNA silencing, in which VPg acts as an accessory factor for HC-Pro (silencing suppressor protein) and promotes disturbance of siRNA and microRNA processing in the nucleus (Kasschau and Carrington, 1998; Rajamäki and Valkonen, 2009).

## Antiviral roles of plant argonautes in translation repression and virus countermeasures

**Figure 2:**
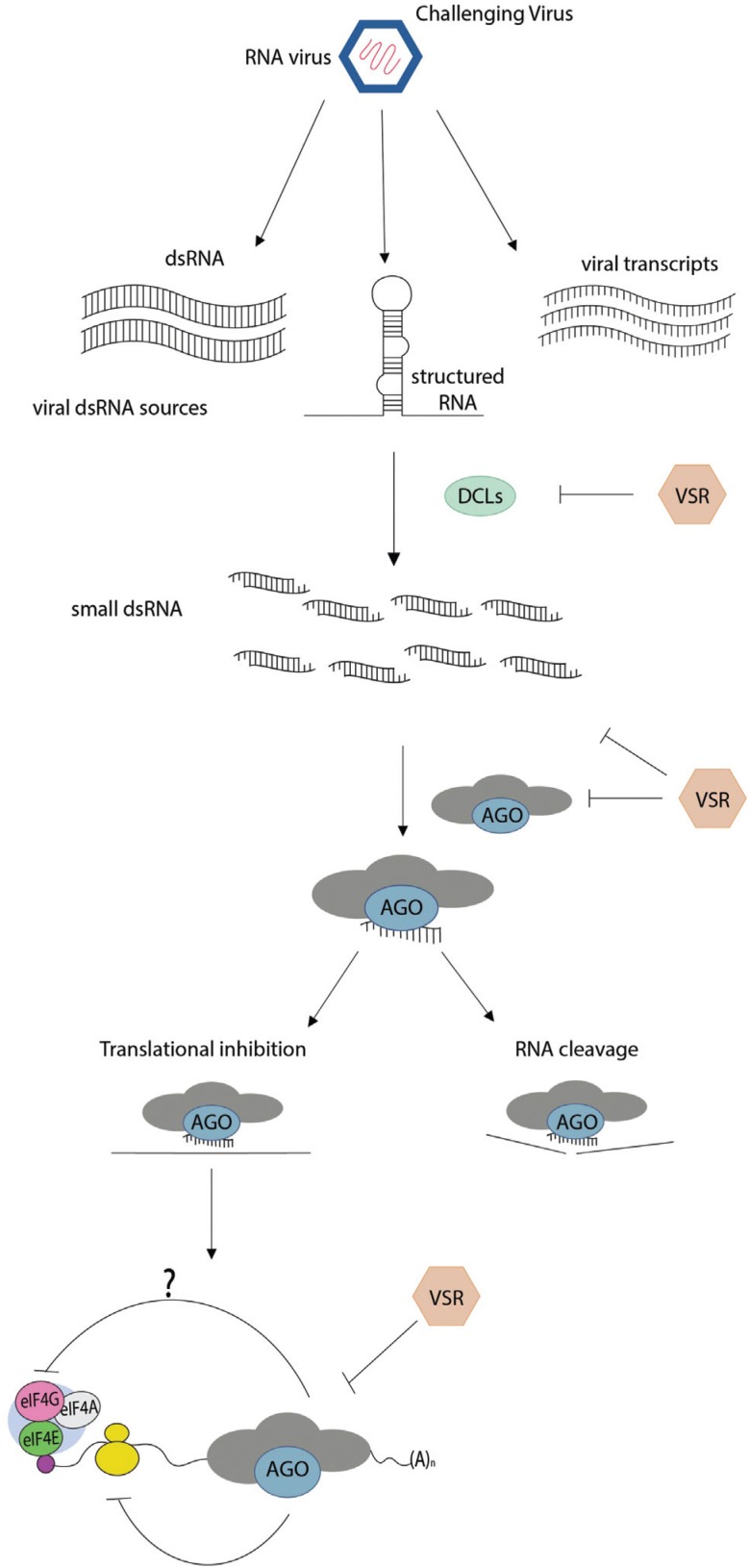
An integrating overview of RNA silencing and AGO-mediated translational repression of mRNA targets. RNA silencing is launched by viral double-stranded RNAs (dsRNAs) from different sources, which are processed into small interfering RNA molecules of 21–24 nucleotide (nt) by Dicer-like (DCL) proteins. Subsequently one strand of the siRNA duplexes is loaded into RNA-induced silencing complexes (RISCs) harboring an argonaute (AGO) effector protein. In light of post-transcriptional gene silencing (PTGS), AGO/sRNA complexes trigger viral RNA cleavage or translational repression. Although unclear, translation repression directed by sRNAs in plants seems to rely on AGO activity, which may act targeting ribosome assembly, interfering with 48S initiation complex formation, or translational initiation factors (eIFs). The AGO-RISC complex is further capable of repressing translation by preventing translation elongation or ribosomal recruitment. Viral suppressors of RNA silencing (VSRs) interfere in multiple steps of the antiviral RNA silencing pathway, including dicing inhibition, viral RNA loading, AGO inactivation and suppression of its translational inhibitory activity.

Argonautes (AGOs) are the effector proteins functioning in eukaryotic RNA silencing pathways (Carbonell and Carrington, 2015; Fang and Qi, 2016), a sequence-specific process that serves two main functions: regulation of gene expression and defense against pathogens (Mandadi and Scholthof, 2013; Bologna and Voinnet, 2014; Nicaise, 2014; Zhang *et al.*, 2015; Wang and Chekanova, 2016). RNA silencing is triggered by the presence of double-stranded RNA (dsRNA), which is processed into small RNA (sRNA) molecules of 21–24 nucleotide (nt) by RNase III-type enzymes called Dicer, or Dicer-like (DCL) in plants (Figure 2; Krol *et al.*, 2010). Upon processing, one strand of the sRNA duplexes is incorporated into RNA-induced silencing complexes (RISCs), whose key catalytic component corresponds to one member of the AGO protein family. Once integrated into the RISC, sRNAs guide the sequence-specific inactivation of the targeted RNA or DNA (Kamthan *et al.*, 2015). The mechanisms of action of AGO/sRNA complexes at the RNA level include mRNA cleavage or translational repression (post-transcriptional gene silencing, PTGS; Figure 2), whereas, at the DNA level, they involve DNA and/or histone methylation and subsequent transcriptional gene silencing (TGS) (Martinez de Alba *et al.*, 2013).

Translational repression guided by sRNAs has been best studied in fly and mammalian cells and is mediated by imperfect base pairing of microRNAs (miRNAs) to target mRNAs (Wilczynska and Bushell, 2015). In animal cells, miRNAs normally bind to the 3’-untranslated region (3’UTR) of target mRNAs and direct not only translation repression but also mRNA destabilization, which are initiated by deadenylation/decapping enzymes. Both processes require association of AGO proteins with proteins containing glycine-tryptophan (GW/WG) motifs, such as members of the GW182 family (Fukaya and Tomari, 2012; Pfaff and Meister, 2013). In plants, early studies have shown that miRNAs display a high degree of sequence complementarity to their target mRNAs and they guide cleavage of target RNAs through endonucleolytic activity of AGO1 (Tang *et al.*, 2003; Baumberger and Baulcombe, 2005). This led to the assumption that RNA cleavage is the major mode of action of plant miRNAs (Jones-Rhoades *et al.*, 2006; Huntzinger and Izaurralde 2011). However, recent reports suggest that plant miRNAs mediate not only the cleavage of the target but also a concurrent translation repression (Brodersen *et al.*, 2008; Lanet *et al.*, 2009; Yang *et al.*, 2012; Iwakawa and Tomari, 2013; Li *et al.*, 2013). Evidence that miRNAs repress translation in plants emerged from the examination of protein accumulation from miRNA-targeted genes (Aukerman and Sakai, 2003; Chen, 2004; Gandikota *et al.*, 2007). In these studies, plant miRNAs were found to exert disproportionate effects on target gene expression at mRNA versus protein levels. Furthermore, mutations in a number of genes, including the P body component VARICOSE (VCS) and ALTERED MERISTEM PROGRAM1 (AMP1), an integral membrane protein associated with endoplasmic reticulum (ER) and AGO1, impair miRNA-mediated target repression at the protein but not at the mRNA level (Brodersen *et al.*, 2008; Li *et al.*, 2013). Additional evidence for the miRNA-mediated translational repression in plants came from the observation that AGO1 and several miRNA are associated with polysomes (Llave *et al.*, 2002).

AGO1, AGO2 and AGO10 have been implicated in translation repression (Brodersen *et al.*, 2008; Lanet *et al.*, 2009; Fátyol *et al.*, 2016). Mutations in AGO1 and AGO10 genes impair miRNA-mediated target repression at the protein but not at the mRNA level (Brodersen *et al.*, 2008). Furthermore, by using an *in vitro* system prepared from plant cultured cells, AtAGO1 has been shown to have the ability to repress translation initiation even when the cleavage of mRNA targets was blocked by introducing central mismatches in the miRNA-target pairing region or mutation of the catalytic core of AtAGO1 (Iwakawa and Tomari, 2013). Recently, Fátyol *et al.* (2016) showed, using a sensitive transient *in vivo* reporter system, that AGO2 is capable of exerting translational repression in various miRNA target site constellations (Open reading frame – ORF, 3’UTR).

The mechanism of AGO-mediated translational repression in plant cells is less clear than in animal cells. Although plant cells apparently lack orthologs of GW182 (Huntzinger and Izaurralde 2011), which are proteins containing GW/WG motifs required for AGO-mediated translation suppression in animal cells (Fukaya and Tomari, 2012; Pfaff and Meister, 2013), the Arabidopsis SUO protein (a large protein with two C-terminal GW motifs) is required for miRNA directed translation repression and may serve as a functional analog of the GW182 proteins (Yang *et al.*, 2012). In line with the notion that plants likely lack orthologs of GW182, deadenylation of the mRNAs has not been observed in tobacco cell lysates (Iwakawa and Tomari, 2013). Binding of miRNAs is not only restricted to the 3’UTR of the mRNAs but also occurs in the 5’UTR and even in the ORF (Iwakawa and Tomari, 2013; Fátyol *et al.*, 2016). A detailed *in vitro* study revealed that several distinct types of translation repression could be mediated by plant miRNAs depending on the position of the target sites (Iwakawa and Tomari, 2013). When target sites reside in the 3’UTR of the mRNAs, AtAGO1-RISC is capable of repressing translation initiation by interfering with 48S initiation complex formation, a mechanism similar to that observed in animal cells (Figure 2). Binding of miRNAs to targets within the ORF functions differently by preventing translation elongation (Iwakawa and Tomari, 2013). When extensively complementary target sites reside in the 5’UTR, AtAGO1-RISC can sterically hinder ribosomal recruitment (Iwakawa and Tomari, 2013).

Antiviral RNA silencing is triggered by highly structured viral ssRNA or dsRNA, which are recognized and processed by DCLs to produce viral small interfering RNAs (vsiRNAs) that are subsequently incorporated into antiviral RISCs (Mallory and Vaucheret, 2010). Antiviral AGOs associate with vsiRNAs and target complementary viral RNAs for degradation through endonucleolytic cleavage (slicing) and/or for translational arrest, transcriptionally repress complementary viral DNA through hypermethylation, or regulate host gene expression to promote defense (Szittya and Burgyan, 2013). These processes can result in a phenomenon known as recovery, whereby the plant silences viral gene expression and recovers from viral symptoms. The targeting of viral RNAs is thought to largely involve RNA cleavage. Nevertheless, recent studies have identified AGO-mediated translation repression as an additional RNA silencing mechanism against plant viruses (Bhattacharjee *et al.*, 2009; Ghoshal and Sanfaçon, 2014; Karran and Sanfaçon, 2014; Ma *et al.*, 2015).

Translational repression of viral mRNAs was first observed in association with the defense response activated by the interaction between a dominant resistance gene and a viral elicitor (Bhattacharjee *et al.*, 2009). Co-expression of a resistance protein with nucleotide-binding (NB) and leucine-rich repeat (LRR) domain (NB–LRR) and its cognate viral effector results in an antiviral response that inhibits the translation of virus-encoded proteins in *Nicotiana benthamiana* (Bhattacharjee *et al.*, 2009). Both the translational repression of viral transcripts and NB-LRR-mediated virus resistance were impaired by the downregulation of Argonaute 4-like genes. These results suggest that AGO proteins are involved in the specific translational control of viral transcripts in virus resistance mediated by NB–LRR proteins (Bhattacharjee *et al.*, 2009). Translation inhibition was also observed in a study with *Tobacco rattle virus* (TRV) in Arabidopsis (Ma *et al.*, 2015). In this study, recovered plants showed reduced association of TRV RNAs with ribosomes and an increase in the formation of RNA processing bodies (PBs). Another example of AGO-dependent translational repression mechanism was observed in *N. benthamiana* plants infected with *Tomato ringspot virus* (ToRSV) (Ghoshal and Sanfaçon, 2014). In this interaction, symptom recovery follows an initial symptomatic systemic infection. These authors also showed that the recovery of ToRSV-infected plants is associated with a reduction in the steady-state levels of viral proteins and decreased translation of the corresponding viral RNA. *In vivo* labeling experiments revealed efficient synthesis of the RNA2-encoded coat protein (CP) early in infection, but reduced RNA2 translation later in infection. Additionally, neither recovery nor the reduction of RNA2 translation were observed in plants silenced for AGO1, suggesting that AGO1 plays a role in the translational repression mechanism targeting ToRSV (Ghoshal and Sanfaçon 2014).

As a counter-defense strategy against antiviral RNA silencing mechanism, most plant viruses have evolved specialized proteins known as viral suppressors of RNA silencing (VSRs), which disrupt various steps of the silencing pathway (Burgyán and Havelda 2011; Pumplin and Voinnet, 2013; Csorba *et al.*, 2015). AGO proteins are preferred targets of VSRs at multiple levels (Figure 2). Among the well-characterized VSRs, some of them (e.g., the tombusvirus p19 and the potyvirus HC-Pro proteins) directly bind and sequester vsiRNA duplexes away from antiviral AGOs, preventing their loading into the RISC (Vargason *et al.*, 2003; Ye *et al.*, 2003; Csorba *et al.*, 2015; Garcia-Ruiz *et al.*, 2015). VSRs can also prevent AGO association with vsiRNAs by promoting AGO degradation, as observed for polerovirus and enamovirus. P0 proteins destabilize AGO1 through an F-box-like domain and induce subsequent degradation through the autophagy pathway (Baumberger *et al.*, 2007; Bortolamiol *et al.*, 2007; Csorba *et al.*, 2010; Derrien *et al.*, 2012; Fusaro *et al.*, 2012). Likewise, the silencing suppressor P25 of *Potato virus X* interacts with AGO1 and mediates its degradation through the proteasome pathway (Chiu *et al.*, 2010).

VSRs can also block vsiRNA-programmed AGOs. The cucumovirus 2b protein interacts directly with AGO1, and this interaction occurs primarily on one surface of the PAZ domain and part of the PIWI domain of AGO1 (Zhang *et al.*, 2006). Consistent with this interaction, 2b specifically inhibits the AGO1 slicing activity in RISC (Zhang *et al.*, 2006). Some VSRs, including the ipomovirus P1, carmovirus p38 and nepovirus CP proteins, contain WG/GW motifs that mimic AGO1-interacting cellular proteins (Azevedo *et al.*, 2010; Giner *et al.*, 2010; Szabo *et al.*, 2012; Zhang *et al.*, 2012; Karran and Sanfaçon, 2014). P1 protein from *Sweet potato mild mottle virus* (SPMMV) targets loaded AGO1 and inhibits the si/miRNA-programmed RISC activity. The suppressor/binding activities are localized at the N-terminal half of P1, a region containing three WG/GW motifs (Giner *et al.*, 2010). The importance of the Glycine-Tryptophan (GW) motifs in AGO1 binding and suppression activity was further demonstrated when *Sweet potato feathery mottle virus* (SPFMV) P1, which did not have any silencing suppressor activity, was converted into a VSR by including two additional WG/GW motifs (Szabo *et al.*, 2012). P38 protein from *Turnip crinkle virus* (TCV) physically interacts through GW repetitive motifs with unloaded Arabidopsis AGO1 (Azevedo *et al.*, 2010) or AGO2 (Zhang *et al.*, 2012), suppressing RNA silencing. Another example of VSR that acts through interaction with AGO1 in a WG motif-dependent manner is ToRSV CP protein (Karran and Sanfaçon, 2014). The WG motif within the CP is required for silencing suppression, AGO1 binding, CP mediated AGO1 degradation, suggesting that the ToRSV CP acts as an AGO-hook protein and competes for AGO binding with a plant cellular GW/WG protein involved in translation repression (Karran and Sanfaçon, 2014).

## The translational control branch of the NIK-mediated antiviral signaling

The immune receptor NIK1 [nuclear shuttle protein (NSP)-interacting kinase 1] has a remarkable role in the defense response against begomoviruses. It belongs to the receptor-like kinase (RLK) family of plant receptors, and it was first identified as a virulence target of the begomovirus nuclear shuttle protein (NSP) (Fontes *et al.*, 2004). NSP is encoded by the component B, DNA-B, of bipartite begomoviruses (*Geminiviridae* family) that also encodes the movement protein (MP), both being viral proteins required for systemic infection (Hanley-Bowdoin *et al.*, 2013). The proteins required for replication (Rep and REn), transactivation of viral genes (TrAP), the suppression of RNAi defense functions (TrAP and AC4) and encapsidation of viral DNA (CP) are encoded by the other genomic component, DNA-A. Begomoviruses replicate their genome in the nuclei of infected plants via rolling circle replication. NSP facilitates the traffic of viral DNA from the nucleus to the cytoplasm and acts in concert with MP to move the viral DNA to the adjacent, uninfected cells (Hanley-Bowdoin *et al.*, 2013).

Virus propagation is usually restricted by the activation of the small interfering RNA (siRNA) antiviral machinery and/or salicylic acid (SA) signaling pathway (Nicaise, 2014). In the case of begomoviruses, it has been shown that in addition to encoding suppressors for siRNA-mediated defenses, these viruses enhance their pathogenicity in susceptible hosts by suppressing the antiviral activity of the transmembrane receptor NIK1 by the viral NSP (Fontes *et al.*, 2004; Santos *et al.*, 2009; Brustolini *et al.*, 2015).

Within the RLK family, NIKs receptors (NIK1, NIK2 and NIK3) belong to the subfamily II of leucine-rich repeat (LRR)-RLKs, designated LRRII-RLK group (Shiu and Bleecker, 2001; Dievart and Clark, 2004). NIK1 was identified through two-hybrid screening using the viral protein NSP as bait (Fontes *et al.*, 2004; Mariano *et al.*, 2004). The NSP-NIK1 interaction was further demonstrated by *in vitro* GST pull-down assays and confirmed *in planta* through bimolecular fluorescence complementation (BiFC) assays (Fontes *et al.*, 2004; Brustolini *et al.*, 2015). The NSP–NIK interaction is conserved among begomovirus NSPs and NIK homologues from different hosts. NIK homologs from Arabidopsis, tomato and soybean interact with NSP from *Cabbage leaf curl virus* (CaLCuV) and from tomato-infecting begomoviruses, such as *Tomato golden mosaic virus* (TGMV), *Tomato crinkle leaf yellow virus* (TCrLYV) and *Tomato yellow spot virus* (ToYSV) (Fontes *et al.*, 2004; Mariano *et al.*, 2004; Sakamoto *et al.*, 2012). Using the two-hybrid system in yeast, the NSP-binding site was mapped to an 80 amino acid stretch of the kinase domain (positions 422–502) of NIK1 that encompasses the putative active site for Ser/Thr kinases (subdomain VIb–HrDvKssNxLLD) and the activation loop (subdomain VII–DFGAk/rx, plus subdomain VIII–GtxGyiaPEY) (Fontes *et al.*, 2004).

NSP from CaLCuV acts as a virulence factor to suppress the kinase activity of transmembrane receptor NIKs, suggesting that NIK is involved in antiviral defense response (Fontes *et al.*, 2004). Several lines of evidence further support a NIK role in antiviral defense. Firstly, loss of *NIK* function in Arabidopsis is linked to an enhanced susceptibility phenotype to infection by a coat protein-less mutant of CaLCuV (Fontes *et al.*, 2004; Carvalho *et al.*, 2008; Santos *et al.*, 2009). In addition, overexpression of *NIK1* from Arabidopsis in tomato plants attenuates symptom development and delays ToYSV infection (Carvalho *et al.*, 2008). Finally, mutations in the activation loop (A-loop) of NIK1 that block its autophosphorylation activity also impair the capacity of NIK1 to elicit a response against begomoviruses (Santos *et al.*, 2009).

As Ser/Thr kinase receptors, NIKs contain all of the 11 conserved subdomains of protein kinases, in addition to specific signatures of serine/threonine kinases in subdomains VIb and VIII (Hanks *et al.*, 1988), including the A-loop, region highly conserved among members of the LRRII-RLK subfamily and other members of the extended LRR-RLK family (Hubbard, 1997; Bellon *et al.*, 1999; Biondi *et al.*, 2002; Yang *et al.*, 2002; Kornev *et al.*, 2006). NIK1 kinase activity has been shown to be dependent on the phosphorylation status of the A-loop (Fontes *et al.*, 2004; Carvalho *et al.*, 2008; Santos *et al.*, 2009). NIK1 is phosphorylated *in vitro* at the conserved positions Thr-474 and Thr-469, and mutations within the A-loop interfere in the NIK1 capacity of autophosphorylation (Santos *et al.*, 2009). Replacement of Thr474 with alanine (T474A) strongly inhibits the autophosphorylation activity. This activity is completely abolished by removing the conserved Gly-473 residue in the T474A mutant to valine (G473V/T474A). In contrast, replacement of Thr-474 with a phosphomimetic aspartate residue increases autophosphorylation activity and results in constitutive activation of a NIK1 mutant receptor that it is no longer inhibited by the begomovirus NSP (Santos *et al.*, 2009). The biological relevance of these findings has been certified by *in vivo* complementation assays. Ectopic expression of T474A defective kinase or G473A/T474A inactive kinase does not complement the *nik1* loss-of-function defect, demonstrating that Thr-474 autophosphorylation is required to transduce a defense response to begomoviruses (Santos *et al.*, 2009). In contrast, ectopic expression of the Arabidopsis phosphomimetic T474D mutant in tomato transgenic lines confers higher level of tolerance to tomato-infecting begomoviruses than expression of an intact *NIK1* receptor (Brustolini *et al.*, 2015). Collectively, these results implicate the phosphorylation at the essential Thr-474 residue within the A-loop as a key regulatory mechanism for NIK activation.

The ribosomal protein L10 (RPL10), isolated through two-hybrid screening by its capacity to bind to the kinase domain of NIK1 (Rocha *et al.*, 2008), acts as a downstream effector of the NIK-mediated antiviral response (Carvalho *et al.*, 2008). Consistent with an RPL10 role in antiviral defense, loss of *RPL10* function recapitulated the *nik1* enhanced susceptibility phenotype to begomovirus infection, as the *rpl10* knockout lines developed similar severe symptoms and displayed similar infection rate as *nik1* (Carvalho *et al.*, 2008; Rocha *et al.*, 2008). The RPL10 protein from Arabidopsis shows sequence similarity with the human L10 protein, also called the QM, and, like QM, displays nucleocytoplasmatic shuttling. In fact, RPL10 is localized in the cytoplasm, but is phosphorylated and redirected to the nucleus by co-expression with NIK1 (Carvalho *et al.*, 2008). Although RPL10 binds to NIK1 *in vitro* and *in vivo*, it is not efficiently phosphorylated by NIK1 *in vitro* and may not serve as a direct NIK1 substrate *in vivo*. Nevertheless, several lines of evidence indicate that the nucleocytoplasmic shuttling of RPL10 is dependent on the phosphorylation status and kinase activity of NIK1. While the defective T474A or the inactive G473A/T474A NIK1 mutants failed to redirect RPL10 to the nuclei of co-transfected cells, expression of the hyperactive T474D mutant increased the efficiency of NIK1-mediated RPL10 nuclear localization in co-transfected cells (Carvalho *et al.*, 2008; Santos *et al.*, 2009). Furthermore, NIK1 does not relocate a phosphorylation-deficient mutant of RPL10 to the nucleus (Carvalho *et al.*, 2008). Finally, mutations in the A-loop similarly affect the NIK1 capacity to mediate a phosphorylation-dependent nuclear relocalization of the RPL10 downstream component and to trigger an antiviral response (Carvalho *et al.*, 2008; Santos *et al.*, 2009). These data suggest that, although RPL10 is not a substrate for NIK1 protein, its nucleocytoplasmic shuttling is regulated by phosphorylation and is dependent on the kinase activity of NIK1, classifying RPL10 as a downstream effector of the NIK1-mediated signaling.

To gain further mechanistic insights into the role of NIK1 in antiviral immunity, the induced and repressed transcriptome by expressing the NIK1 phosphomimetic gain-of-function mutant T474D was assessed in Arabidopsis (Zorzatto *et al.*, 2015). NIK1 constitutive activation does not induce the expression of typical defense marker genes associated to gene silencing, salicylic acid, or PAMP-triggered immunity (PTI) pathways but rather it down-regulates translation-related genes, causing suppression of global *in vivo* translation and decreased loading of host mRNA in actively translating polysomes (PS) fractions. Likewise, induction of T474D expression through a dexamethasone-inducible promoter also impairs global translation, which was correlated with a reduction of both PS and monosome (NPS) fractions, as well as of the RNA content associated with these fractions in the T474D lines. Ectopic expression of T474D controls begomovirus infection, causing symptomless infection, delayed course of infection and reduced accumulation of viral DNA in systemically infected leaves. Additionally, in infected T474D lines, the loading of coat protein viral mRNA in actively translating polysomes is reduced as compared to that of wild type infected lines, suggesting that the translation of viral transcripts is strongly impaired by NIK1-mediated signaling. Thus, begomovirus cannot sustain high levels of viral mRNA translation in the T474D-expressing lines, indicating that suppression of global protein synthesis may effectively protect plant cells against DNA viruses (Zorzatto *et al.*, 2015). Supporting this hypothesis, the T474D-overexpressing tomato transgenic lines are tolerant to the tomato-infecting begomoviruses ToYSV and *Tomato severe rugose virus* (ToSRV) (Brustolini *et al.*, 2015), which display highly divergent genomic sequences and hence are phylogenetically separated within the two major groups of begomoviruses found in Brazil (Albuquerque *et al.*, 2012). In addition, the gain-of-function mutant T474D from Arabidopsis functions similarly in tomato plants, as it causes a general down-regulation of translation machinery-related genes, affects translation in transgenic tomato lines and decreases viral mRNA association with the polysome fractions (Brustolini *et al.*, 2015). Therefore, the enhanced tolerance to tomato-infecting begomovirus displayed by the T474D-expressing lines is associated with the translational control branch of the NIK-mediated antiviral responses. These observations demonstrate the potential of a sustained NIK1-mediated defense pathway to confer broad-spectrum tolerance to begomoviruses in distinct plant species. Nevertheless, in the Arabidopsis homologous system, the level of translational inhibition by the constitutive activation of NIK1 causes stunted growth in transgenic lines grown under short-day conditions, whereas, in tomato, ectopic expression of the T474D mutant does not impact development under greenhouse conditions (Brustolini *et al.*, 2015; Zorzatto *et al.*, 2015). As a possible explanation for this phenotype, tomato plants may not need maximal translational capacity for optimal growth under greenhouse conditions; thereby, the level of translational inhibition mediated by NIK1 activation does not reach a threshold that would impact growth. Additionally or alternatively, the T474D-mediated translational suppression provokes a constant perception of stress in the transgenic lines, which, in turn, promotes acclimation to maintain normal growth under greenhouse conditions. Therefore, the intrinsic capacity of agronomically relevant crops to withstand the deleterious effect from the suppression of global translation is a relevant agronomic trait to be considered for engineering the NIK1-mediated resistance against begomoviruses in crops.

Recent progress towards directly connecting the NIK1-mediated signaling pathway with the downregulation of translational-machinery-related genes includes the isolation of a transcription factor harboring a MYB domain, named L10-INTERACTING MYB DOMAIN-CONTAINING PROTEIN (LIMYB), which interacts with RPL10 in the nucleus of plant cells (Zorzatto *et al.*, 2015). The interaction between LIMYB and RPL10 results in the formation of a transcriptional repressor complex that specifically suppresses the expression of ribosomal protein (RP) genes through the binding of LIMYB on RP gene promoters. This RP down-regulation leads to protein synthesis inhibition and enhanced tolerance to the begomovirus CaLCuV. T474D also down-regulates the expression of the same sub-set of LIMYB-regulated RP genes but requires the LIMYB function to repress RP gene expression. In addition, the loss of LIMYB function releases the repression of translation-related genes and increases susceptibility to CaLCuV infection (Zorzatto *et al.*, 2015). Collectively, these results provide both genetic and biochemical evidence that the LIMYB gene functions as a downstream component of the NIK1-mediated signaling pathway linking NIK1 activation to global translation suppression and tolerance to begomiviruses.

**Figure 3:**
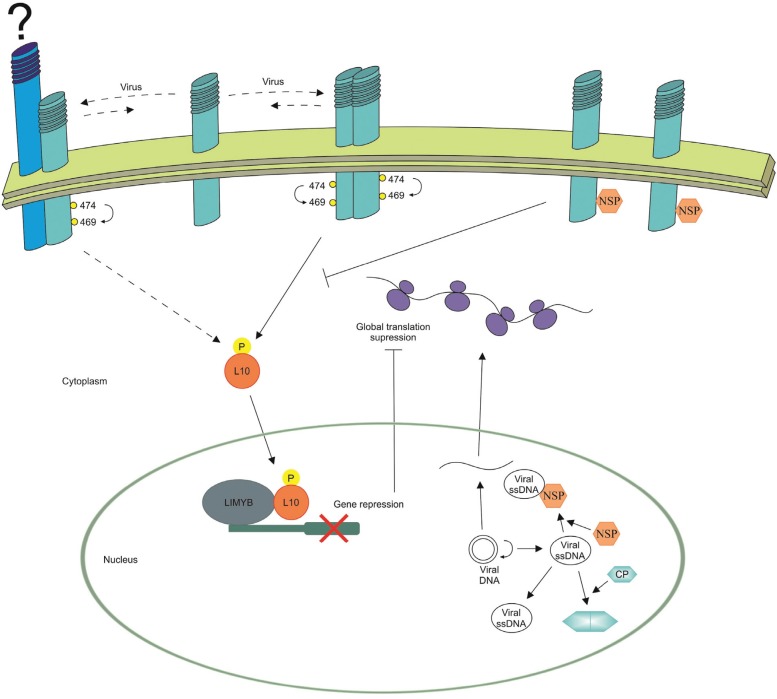
Mechanistic overview of the antiviral defense pathway mediated by NIK1. Upon virus infection, NIK1 forms a homodimer and is activated through transphosphorylation of its kinase domain at the Thr-474 residue. Alternatively, NIK1 binds to an unknown ligand-binding LRR-RLK in a stimulus-dependent manner. The activation of NIK1 triggers the phosphorylation of RPL10 that, in turn, is transported to the nucleus. In the nuclear compartment, RPL10 interacts with LIMYB, which binds to the promoter of ribosomal protein (RP) genes to repress their transcription. As a consequence, a suppression of host global protein synthesis is observed, which also impairs translation of viral mRNA. As a defense countermeasure, NSP of begomoviruses binds and inhibits the NIK1 kinase activity, which impairs the RPL10 phosphorylation. Thus, RPL10 is retained in the cytosol, enhancing begomovirus infection. As begomoviruses have single-stranded circular DNA genomes, they replicate in the nucleus of the infected cells by double-stranded DNA intermediaries, which are also template for transcription of viral mRNAs. NSP binds to nascent viral DNA and facilitates the traffic of viral DNA from the nucleus to the cytoplasm by an unclear mechanism.

Despite the advances in the elucidation of NIK-mediated antiviral signaling pathway, there is a complete lack of information on the critical early event that triggers the NIK1 signaling and transduction, which culminates with the suppression of host global translation as an antiviral response. Recently, a comparison between the transcriptomes induced by begomovirus infection and by expression of the gain-of-function T474D mutant revealed that begomovirus infection is the activating stimulus of NIK1-mediated defense, although the molecular basis for this elicitation is still unknown (Machado *et al.*, 2015; Zorzatto *et al.*, 2015). A mechanistic model for a NIK1-mediated defense signaling pathway and its interaction with the begomovirus NSP is illustrated in Figure 3. Upon begomovirus infection, the extracellular domain of NIK undergoes oligomerization, allowing the intracellular kinase domains to transphosphorylate on a key threonine residue at position 474 (T474) and to activate one another (Santos *et al.*, 2009). Alternatively or additionally, NIK1 may serve as a co-receptor for a defense-signaling cascade and interacts with an unidentified ligand-dependent LRR-RLK receptor in response to virus infection. The phosphorylation-dependent activation of NIK leads to the phosphorylation of RPL10 and the phosphorylated RPL10 is translocated to the nucleus, where it interacts with LIMYB to fully down-regulate translation machinery-related genes, leading to host global translation suppression that affects the translation of the begomovirus mRNAs (Carvalho *et al.*, 2008; Zorzatto *et al.*, 2015). Thus, this down-regulation of cytosolic translation underlies at least partially the molecular mechanisms involved in the NIK1-mediated antiviral defense, which can be suppressed by binding of NSP to the NIK1 kinase domain.

## Conclusions

Due to the agronomic importance of plant virus as pathogens, the development of antiviral strategies aiming crop protection has been continually on focus. In this context, the identification and characterization of host factors targeted during infection constitute one of the most important goals of the virology research. Due to their limited viral genome-encoded functions, the viruses have developed diverse strategies to hijack the host translation apparatus to quickly and efficiently produce viral proteins. Thus, translation repression has emerged as a plant antiviral defense strategy to impair the translation of viral proteins and could contribute as targets for the development of resistance strategy for virus control. In fact, plant RNA viruses interact tightly with the host protein synthesis machinery such that host translation initiation factor-encoding genes can function as recessive resistance genes. Furthermore, the translational repression activity of the effector AGO has been recently demonstrated to play a role in the antiviral RNA silencing mechanism. Finally, as a new paradigm in plant antiviral immunity, the activation of the immune receptor NIK1-mediated suppression of translation has been demonstrated to be effective in controlling begomovirus infections. These examples substantiate the notion that impairing viral mRNA translation (specifically or globally) constitutes a promising strategy for plant protection against viruses.
